# Dichloroisocoumarins with Potential Anti-Inflammatory Activity from the Mangrove Endophytic Fungus *Ascomycota* sp. CYSK-4

**DOI:** 10.3390/md16020054

**Published:** 2018-02-09

**Authors:** Yan Chen, Zhaoming Liu, Hongju Liu, Yahong Pan, Jing Li, Lan Liu, Zhigang She

**Affiliations:** 1 School of Marine Sciences, Sun Yat-sen University, Guangzhou 510006, China; chenyan27@mail2.sysu.edu.cn; 2 School of Chemistry, Sun Yat-Sen University, Guangzhou 510275, China; liuzhaom@mail2.sysu.edu.cn (Z.L.); liuhj8@mail2.sysu.edu.cn (H.L.); pan16a@126.com (Y.P.); 3 School of Pharmacy, Guangdong Medical University, Dongguan 523808, China; lijinggdmu@yahoo.com; 4 South China Sea Bio-Resource Exploitation and Utilization Collaborative Innovation Center, Sun Yat-Sen University, Guangzhou 510275, China

**Keywords:** isocoumarin, endophytic fungus, *Pluchea indica*, *Ascomycota* sp., anti-inflammatory, antibacterial

## Abstract

Three new isocoumarins—dichlorodiaportintone (**1**), desmethyldichlorodiaportintone (**2**) and desmethyldichlorodiaportinol (**3**)—as well as six known analogues (**4–9**) were isolated from the culture of the mangrove endophytic fungus *Ascomycota* sp. CYSK-4 from *Pluchea indica*. Their structures were elucidated by analysis of spectroscopic data. The absolute configuration of compounds **1** and **2** were determined by the modified Mosher’s method. Compound **2** showed significant anti-inflammatory activity by inhibiting the production of NO in LPS-induced RAW 264.7 cells with IC_50_ value of 15.8 μM, while compounds **1**, **5**, and **6** exhibited weak activities with IC_50_ values of 41.5, 33.6, and 67.2 μM, respectively. In addition, compounds **1**, **5**, and **6** showed antibacterial effects against *Staphylococcus aureus*, *Bacillus subtilis*, *Escherichia coli*, *Klebsiella pneumoniae*, and *Acinetobacter calcoaceticus* with the MIC values in the range of 25–50 μg·mL^−1^.

## 1. Introduction

**Figure 1:**
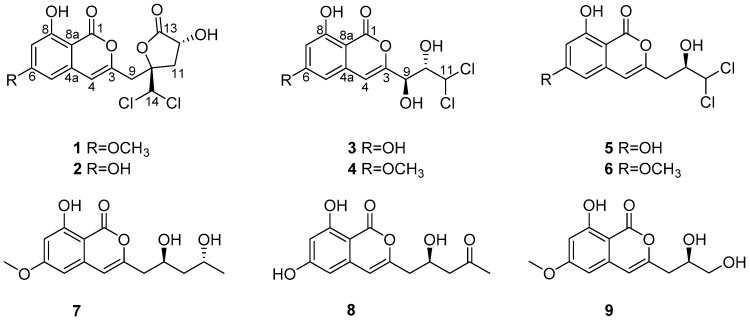
The structures of compounds **1**–**9**.

Fungi in the genus *Ascomycota* sp. produce various structurally novel metabolites, including ascomindones A–C and ascomfurans A–B [1], ascomycotin A [2], wortmannilactone E [3], orsellinic acid [4], isosclerone [5], and chaetocyclinone [6]. Furthermore, most of these compounds possess a wide range of biological activities. For example, polyketone ascomindones A exhibited more potent capacity in scavenging DPPH radical [1]. Diphenyl ether barceloneic acid A showed modest inhibition of FPTase enzyme [7]. As part of our ongoing investigation on bioactive natural products from mangrove-derived fungi, an endophytic fungus *Ascomycota* sp. CYSK-4, which was isolated from the healthy branch of the marine semimangrove *Pluchea indica*, attracted our attention because an EtOAc extract of the fungal culture exhibited significant anti-inflammatory activity. Bioassay-guided fractionation of the EtOAc extract led to the isolation of three new isocoumarin derivatives, compounds **1**–**3**, together with six known isocoumarin analogues **4–9** (Figure 1). In the in vitro assays, compounds **1**–**2** and **5**–**6** showed inhibitory activities against the lipopolysaccharides (LPS) induced nitric oxide (NO) production in murine macrophage RAW 264.7. Herein, the details of the isolation, structural elucidation and anti-inflammatory evaluation of these compounds were reported.

## 2. Results

**Figure 2:**
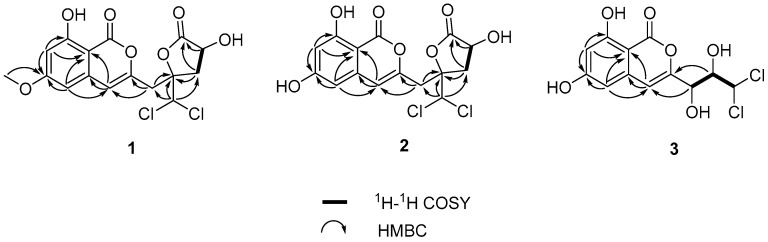
Key COSY and HMBC correlations of compounds **1**–**3**.

Compound **1** was obtained as an amorphous solid. Its molecular formula was established as C_16_H_14_O_7_Cl_2_ based on the HRESIMS and NMR data, containing two Cl-atoms and implying nine indices of hydrogen deficiency. The ^1^H NMR data of **1** (Table 1) displayed signals of two aromatic protons [*δ*_H_ 6.51 (1H, d, *J* = 2.2 Hz); 6.62 (1H, d, *J* = 2.2 Hz)], one olefin proton [6.68 (1H, s)], two methylenes [*δ*_H_ 3.33 (2H, d, *J* = 6.0 Hz); 3.04 (1H, dd, *J* = 9.5, 14.7 Hz); 2.45 (1H, dd, *J* = 6.9, 14.7 Hz)], two methines [*δ*_H_ 4.75 (1H, dd, *J* = 6.9, 9.5 Hz); 6.52 (1H, s)], and one methoxy group at *δ*_H_ 3.92 (1H, s). The ^13^C NMR data of **1** (Table 2) exhibited 16 carbon resonances assignable to one methyl, two methylenes, two sp^3^ and three sp^2^ methines, six quaternary carbons and two carbonyl carbons. These spectroscopic features suggested that **1** belong to the isocoumarin class [8,9,10]. Analysis of the ^1^H-^1^H COSY spectrum (Figure 2) suggested the presence of one independent spin system H_2_-11/H-12. Together with the HMBC cross-peaks (Figure 2) of H-12/C-13; H-11/C-10, C-13; H-9/C-3, C-10; H-14/C-9, C-10, and C-11 indicated the side chain of isocaproicacid moiety location at C-3. The chemical shift of H-14 (*δ*_H_ 6.52) indicated the dichloro substitution at C-14 [8]. Apart from a carbonyl group and isocoumarin group, the remaining one indices of hydrogen deficiency was proved to be *α*-hydroxyl-*γ*-lactone ring. The chemical shift of quaternary carbon *δ*_C_ 86.0 (C-10) confirmed the ring bridging C-10 and C-13. Moreover, the methoxy group was placed to C-6 based on the HMBC correlation of its proton to C-6. Thus, the constitution of **1** was established (Figure 1).

**Figure 3:**
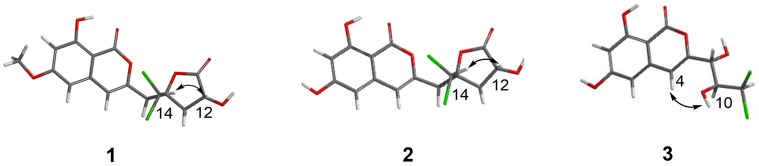
Key NOESY correlations of compounds **1**–**3**.

**Figure 4:**
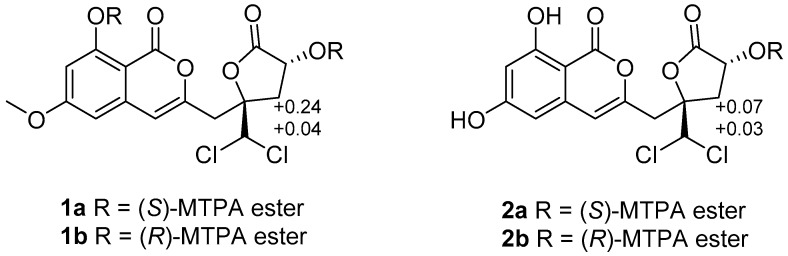
Δ*δ* = *δ_S_* − *δ_R_* values in ppm for the MTPA (Methoxy–trifluoromethyl phenylacetic acid) esters of **1** and **2**.

The relative configuration of **1** was determined by NOESY data (Figure 3). The cross-peak of H-12 and H-14 indicated the *syn* relationship between H-12 and H-14. The absolute configuration of C-12 was further confirmed by the modified Mosher ester method [11]. The (*S*)- and (*R*)-MTPA esters of **1** (**1a** and **1b**) were prepared using (*R*)- and (*S*)-MTPA chloride, respectively. The differences in the ^1^H NMR chemical shifts of **1a** and **1b** were summarized to determine the absolute configuration of this position, which was clearly established as 12*R*. Taking the data discussed above into account, the absolute configuration of **1** was assigned as 10*R*,12*R* (Figure 4). Thus, compound **1** was determined as dichlorodiaportintone (Figure 1).

Compound **2** was isolated as a white amorphous powder, having the molecular formula C_15_H_12_O_7_Cl_2_ based on the HREIMS at *m*/*z* 372.9885 [M − H]^−^. The NMR data (Table 1 and Table 2) resembled those of **1**, except for the disappearence of a methoxy group (*δ*_C_ 56.3, *δ*_H_ 3.92). The NOESY correlation (Figure 3) from H-12 to H-14 proved that compounds **2** and **1** shared the same relative configuration. The absolute configuration at C-12 was also determined as *R* by the modified Mosher’s method. Thus, the absolute configuration of **2** was determined as 10*R*,12*R* (Figure 4). Therefore, the compound **2** was assigned as desmethyldichlorodiaportintone (Figure 1).

**Figure 5:**
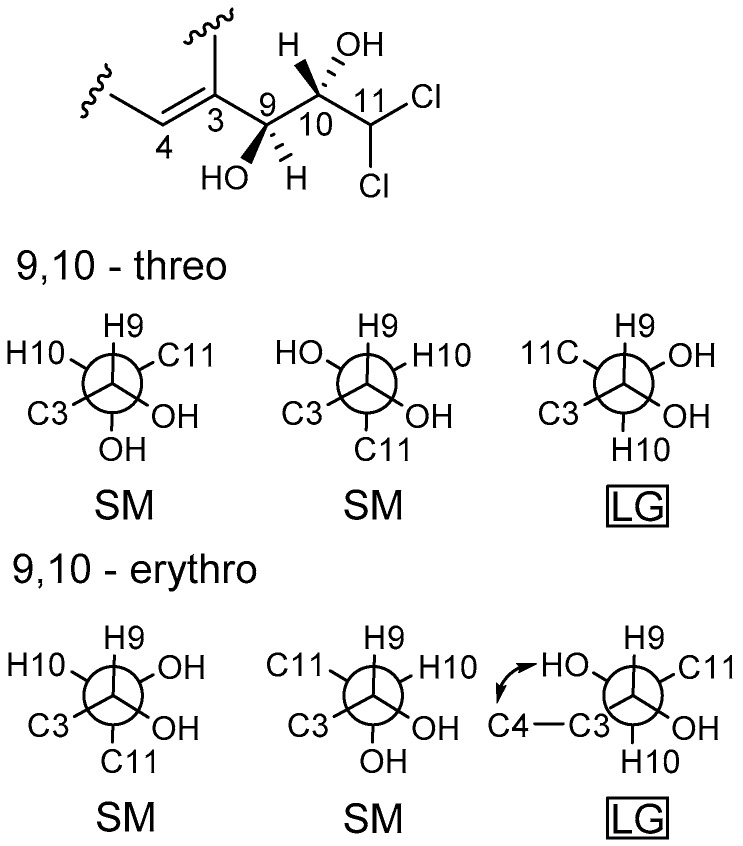
Newman projections for C-9/C-10. Box indicates conformation that consistent with coupling constant. LG: large; SM: small.

Compound **3** was obtained as a white powder. The molecular formula was determined as C_12_H_10_O_6_Cl_2_ by HRESIMS. The ^1^H NMR spectrum (Figure S18) of **3** showed signals for a 1,2,3,5-tetrasubstituted aromatic unit [*δ*_H_ 6.44 (1H, d, *J* = 2.1 Hz) and *δ*_H_ 6.51 (1H, d, *J* = 2.1 Hz)], one methine signal at *δ*_H_ 6.43 (1H, d, *J* = 1.6 Hz), two oxygenated methine signals at *δ*_H_ 4.38 (1H, dd, *J* = 1.6, 8.8 Hz) and *δ*_H_ 4.28 (1H, d, *J* = 8.8 Hz). The ^13^C NMR spectrum (Figure S19) exhibited 13 carbon signals, indicating a carbonyl carbon, three methines, eight aromatic carbons. The ^1^H and ^13^C NMR spectra (Table 1 and Table 2) of **3** were similar to those of dichlorodiaportinol A (**4**), except for the absence of the methoxy group (*δ*_C_ 56.3, *δ*_H_ 3.90) at C-6 in **3**. The structure of **3** was also confirmed using ^1^H-^1^H COSY and HMBC spectra (Figure 2). The configuration of two stereocenters (C-9 and C-10) were determined by coupling constants and NOESY experiments (Figure 3). Protons H-9 and H-10 displayed a large coupling constant (^3^*J*_H-9,H-10_ = 8.8 Hz), indicating them to be in an anti configuration. This allowed for only two of the six possible relative configuration for C-9 and C-10 could be satisfied. As well as the NOE correlation of H-4/OH-10 in DMSO, the relative configuration of C-9 and C-10 was unambiguously determined as 9R* and 10S* (Figure 5) and named desmethyldichlorodiaportinol (Figure 1).

The other known compounds were identified as dichlorodiaportinol (**4**) [9], desmethyldichlorodiaportin (**5**) [8], dichlorodiaportin (**6**) [10], mucorisocoumarin B (**7**) [12], citroisocoumarin (**8**) [13], and diaportinol (**9**) [11] by comparison with NMR data in the literature.

The anti-inflammatory activities of all compounds were evaluated against nitric oxide (NO) production in the lipopolysaccharide (LPS)-stimulated mouse macrophage RAW 264.7. The results suggest that the compound **2** showed potent inhibitory activities with IC_50_ value of 15.8 μM, and compounds **1**, **5**, and **6** exhibited weak inhibitory activity in comparison with the indomethacin (the positive control, IC_50_ = 37.5 μM). Other compounds showed no inhibitory effect (IC_50_ > 100 μM) (Table 3). All compounds showed no cytotoxic effect at the tested concentration. Compounds **2** and **5** which have a hydroxyl group at C-6 showed batter than compounds **1** and **6** with a methoxy group at C-6. The structure-activity relationships of these dichloroisocoumarins indicated that a hydroxyl group was more significance than a methoxy group on anti-inflammatory activity. Isocoumarins were previously reported to have radical scavenging and antioxidant [14], anti-HIV [15], antimicrobial [16], anti-*γ*-secretase [17], antitumor [18], immunomodulatory [19], antifungal [20], toxicity to zebrafish embryos [13], and *α*-glucosidase inhibitory activities [21]. This is the first report of anti-inflammatory activity of dichloroisocoumarins.

The antibacterial activities of the isolated compounds **1**–**9** against two Gram-positive bacteria (*Staphylococcus aureus* and *Bacillus subtilis*) and three Gram-negative bacteria (*Escherichia coli*, *Klebsiella pneumoniae*, and *Acinetobacter calcoaceticus*) were tested (Table 4). Compounds **5** and **6** showed antibacterial activities with the MIC values between 25 and 50 μg·mL^–1^ against *S. aureus*, *B. subtilis*, *E. coli*, *K. pneumoniae*, and *A. calcoaceticus.* Compound **1** exhibited antibacterial activities with the MIC values at 50 μg·mL^−1^ against *S. aureus*, *E. coli* and *K. pneumoniae.* Other compounds did not exhibit obvious activity at 50 μg·mL^−1^.

## 3. Materials and Methods 

### 3.1. General Experimental Procedures

Optical rotations were measured on an MCP 300 (Anton Paar, Shanghai, China) polarimeter at 25 °C. UV spectra were recorded in MeOH using a PERSEE TU-1900 spectrophotometer (Persee, Beijing, China). IR spectra were recorded on a Nicolet Nexus 670 spectrophotometer (Nicolet, Madison, WI, USA) in KBr discs. NMR spectra were carried out on Bruker Avance 400 spectrometer (^1^H 400 MHz, ^13^C 100 MHz) (Bruker Bio Spin Corporation, Bellerica, MA, USA) and Bruker Avance 500 spectrometer (^1^H 500 MHz, ^13^C 125 MHz) (Bruker Bio Spin Corporation, Bellerica, MA, USA). ESIMS spectra were measured on a Finnigan LCQ-DECA mass spectrometer (Finnigan, Beijing, China), and HRESIMS spectra were obtained on a Thermo Fisher Scientic Q-TOF mass spectrometer (Thermo Fisher Scientific, Waltham, MA, USA). Column chromatography (CC) was conducted using silica gel (200–300 mesh, Qingdao Marine Chemical Factory, Qingdao, China) and Sephadex LH-20 (Amersham Pharmacia, Piscataway, NJ, USA). Thin-layer chromatography (TLC) was performed on silica gel plates (Qingdao Huang Hai Chemical Group Co., G60, F-254, Qingdao, China).

### 3.2. Fungal Material and Fermentation

The fungus CYSK-4 used in this study was isolated from healthy branch of the marine semimangrove *Pluchea indica*, which was collected in July 2015 from Shankou Mangrove Nature Reserve in Guangxi Province, China. It was obtained using the standard protocol for isolation [21]. Initially, the plant tissue was washed with sterile water and surface-sterilized in a 200 mL beaker with 75% ethanol for 1 min. This was followed by dipping the sample into 5% sodium hypochlorite for 1 min, then the plant parts were rinsed with sterile water, cut into 3 mm sections, and plated on PDA with penicillin (100 units per mL) and streptomycin (0.08 mg·mL^−1^). The plates were incubated at 25 ± 1 °C. The endophytic fungal strains were isolated by routine microbiological methods. The fungal isolates were numbered and stored at 4 °C in triplicate on PDA slants. Fungal identification was carried out using a molecular biological protocol by DNA amplification and sequencing of the ITS region [22]. The sequence data obtained from the fungal strain have been deposited at Gen Bank with accession no. MG571637. A BLAST search result showed that the sequence was the most similar (99%) to the sequence of *Ascomycota* sp. (compared to KT240142.1 EF060747.1). A voucher strain was deposited in the Guangdong Microbial Culture Center under patent depository number GDMCC 60100. The fungus *Ascomycota* sp. CYSK-4 was cultured on autoclaved rice solid-substrate medium (60 × 500 mL Erlenmeyer flasks, each containing 50 g rice and 50 mL 3‰ of saline water) for 30 days at room temperature.

### 3.3. Extraction and Isolation

Following incubation. The mycelia and solid rice medium were extracted three times with EtOAc. The extract was evaporated under reduced pressure to yield 60 g of residue. The residue was subjected to a silica gel column chromatography, eluting with a gradient of petroleum ether/EtOAc from 1:0 to 0:1, to obtain 36 fractions. Fraction 8 (120 mg) was subjected to silica gel CC (CH_2_Cl_2_/MeOH *v*/*v*, 98:2) to yield compounds **1** (8.2 mg), **3** (3.8 mg), and **7** (2.5 mg). Fraction 12 (90 mg) was applied to Sephadex LH-20 CC (CH_2_Cl_2_/MeOH *v*/*v*, 1:1) to give compounds **2** (5.1 mg) and **5** (3.4 mg). Fraction 16 (68 mg) was purified by Sephadex LH-20 CC (CH_2_Cl_2_/MeOH *v*/*v*, 1:1) and silica gel CC (CH_2_Cl_2_/MeOH *v*/*v*, 88:12) afford **4** (4.6 mg) and **8** (2.2 mg). Fraction 18 (62 mg) was chromatographed on Sephadex LH-20 CC (100% MeOH) to obtain compound **6** (1.8 mg). Fraction 20 was separated by silica gel CC (CH_2_Cl_2_/MeOH *v*/*v*, 86:14) to give subfraction fraction 20.2, which was purified by Sephadex LH-20 CC (CH_2_Cl_2_/MeOH *v*/*v*, 1:1) to yield **9** (2 mg).

#### 3.3.1. Dichlorodiaportintone (**1**)

Amorphous solid; [*α*${]}_{25}^{D}$ = +11.9 (*c* 0.12, acetone); UV (MeOH) *λ*_max_ (log *ε*): 332 (3.78), 280 (3.87), 245 (4.61) nm; IR (KBr) *ν*_max_: 3319, 1800, 1681, 1621, 1386, 1238, 1209, 1167, 856, 795, 712 cm^−1^; HRESIMS *m/z* 387.0041 [M − H]^–^ (calcd. for C_16_H_13_O_7_Cl_2_, 387.0048); ^1^H NMR (500 MHz, acetone-*d*_6_) data, see Table 1; ^13^C NMR (125 MHz, acetone-*d*_6_) data, see Table 2.

#### 3.3.2. Desmethyldichlorodiaportintone (**2**)

White amorphous powder; [*α*${]}_{25}^{D}$ = +6.9 (*с* 0.06, acetone); UV (MeOH) *λ*_max_ (log *ε*): 331 (3.85), 262 (4.20), 246 (4.44) nm; IR (KBr) *ν*_max_: 3232, 1781, 1679, 1630, 1455, 1372, 1248, 1166, 1071, 781 cm^−1^; HRESIMS *m/z* 372.9885 [M − H]^−^ (calcd. for C_15_H_11_O_7_Cl_2_, 372.9887); ^1^H NMR (500 MHz, acetone-*d*_6_) data, see Table 1; ^13^C NMR (125 MHz, acetone-*d*_6_) data, see Table 2.

#### 3.3.3. Desmethyldichlorodiaportinol (**3**)

White powder; [*α*${]}_{25}^{D}$ = +18.3 (*с* 0.1, acetone); UV (MeOH) λ_max_ (log *ε*): 329 (3.62), 268 (3.65), 245 (3.26) nm; IR (KBr) *ν*_max_: 3543, 3461, 3232, 1683, 1631, 1497, 1399, 1293, 1242, 1188, 1071, 1038, 837, 703 cm^−1^; HRESIMS *m/z* 318.9782 [M − H]^−^ (calcd. for C_12_H_9_O_6_Cl_2_, 318.9782); ^1^H NMR (500 MHz, acetone-*d*_6_) data, see Table 1; ^13^C NMR (125 MHz, acetone-*d*_6_) data, see Table 2.

#### 3.3.4. Preparation of the (*S*)- and (*R*)-MTPA Esters **1a** and **1b**

Compound **1** (1 mg) was treated with (*R*)-MTPACl (10 μL) and pyridine (0.5 mL). The mixture was reacted at room temperature for 24 h. The solution was extracted with 5 mL of CH_2_Cl_2_, and the organic phase was concentrated under reduced pressure. The residue was purified by preparative TLC (CH_2_Cl_2_) to yield the (*S*)-MTPA ester **1a** (0.8 mg). In a similar way, (*R*)-MTPA ester **1b** (0.5 mg) was obtained from compound **1** (1 mg) reacted with (*S*)-MTPACl (10 μL).

(*S*)-MTPA ester **1a**: ^1^H NMR (acetone-*d*_6_, 400 MHz) *δ*_H_: 6.74 (1H, s, H-4), 6.65 (1H, s, H-14), 7.12 (1H, d, *J* = 2.2 Hz, H-5), 6.72 (1H, d, *J* = 2.2 Hz, H-7), 5.95 (1H, dd, *J* = 8.0, 10.3 Hz, H-12), 3.98 (3H, s, 6-OCH_3_), 3.76 (3H, s, OCH_3_-MTPA), 3.52 (3H, s, OCH_3_-MTPA), 3.43 (2H, d, *J* = 4.6 Hz, H-9), 3.33 (1H, dd, *J* = 7.8, 15.0 Hz, H-11a), 2.85 (1H, dd, *J* = 10.4, 15.0 Hz, H-11b). ESIMS *m/z* 821.0 [M + 1]^+^.

(*R*)-MTPA ester **1b**: ^1^H NMR (acetone-*d*_6_, 400 MHz) *δ*_H_: 6.65 (1H, s, H-4), 6.63 (1H, s, H-14), 6.57 (1H, d, *J* = 2.2 Hz, H-5), 6.52 (1H, d, *J* = 2.2 Hz, H-7), 6.03 (1H, dd, *J* = 8.0, 10.3 Hz, H-12), 3.92 (3H, s, 6-OCH_3_), 3.58 (3H, s, OCH_3_-MTPA), 3.52 (3H, s, OCH_3_-MTPA), 3.38 (2H, d, *J* = 4.6 Hz, H-9), 3.29 (1H, dd, *J* = 7.8, 15.0 Hz, H-11a), 2.61 (1H, dd, *J* = 10.4, 15.0 Hz, H-11b). ESIMS *m/z* 821.0 [M + 1]^+^.

#### 3.3.5. Preparation of the (*S*)- and (*R*)-MTPA Esters **2a** and **2b**

Following the same way as described for compound **1**, *R*- and *S*- MTPA ester derivatives **2a** and **2b** were prepared from **2**.

(*S*)-MTPA ester **2a**: ^1^H NMR (CHCl_3_, 400 MHz) *δ*_H_: 6.96 (1H, s, H-4), 6.95 (1H, s, H-14), 7.01 (1H, s, H-5), 6.69 (1H, s, H-7), 6.35 (1H, d, *J* = 2.31 Hz, H-12), 3.53 (3H, s, OCH_3_-MTPA), 3.46 (2H, m, H-9), 2.98 (1H, dd, *J* = 10.6, 14.9 Hz, H-11a), 2.55 (1H, dd, *J* = 7.7, 14.9 Hz, H-11b). ESIMS *m/z* 591.0 [M + 1]^+^.

(*R*)-MTPA ester **2b**: ^1^H NMR (CHCl_3_, 400 MHz) *δ*_H_: 6.91 (1H, s, H-4), 6.93 (1H, s, H-14), 7.0 (1H, s, H-5), 6.62 (1H, s, H-7), 6.33 (1H, d, *J* = 2.31 Hz, H-12), 3.53 (3H, s, OCH_3_-MTPA), 3.40 (2H, m, H-9), 2.91 (1H, dd, *J* = 10.6, 14.9 Hz, H-11a), 2.52 (1H, dd, *J* = 7.7, 14.9 Hz, H-11b). ESIMS *m/z* 591.0 [M + 1]^+^.

### 3.4. Nitric Oxide Production Assay

Murine macrophage RAW 264.7 cells purchased from the Shanghai Institutes for Biological Sciences in DMEM (high glucose) medium supplemented with 10% (*v*/*v*) fetal bovine serum, 100 μg·mL^‒1^ penicillin and streptomycin, and 10 mM HEPES at 37 °C in a 5% CO_2_ atmosphere [23]. Cells were pretreated with different samples dissolved in serum-free culture medium containing 0.5% DMSO (10, 5, 2.5, 1.25, and 0.625 μM) for 4 h, followed by stimulation with 1 μg·mL^–1^ LPS for 24 h. Fifty μL of cell culture medium was mixed with 100 μL of Griess reagent I and II and incubated at room temperature for 10 min with horizontal shaking, after which the absorbance at 540 nm was measured in a microplate reader. Indomethacin was used as a positive control and was purchased from Sigma-Aldrich Co. (CAS number: 53-86-1, EINECS number: 200-186-5; Buchs, Switzerland). Wells with DMSO were used as a negative control (final DMSO concentration was 0.1%). The NO production inhibition rate was calculated by the flowing formula:

(1) $$\mathrm{NO}\;\mathrm{production}\;\mathrm{inhibition}\;\mathrm{rate}\;\left(\%\right)=\frac{\mathrm{LPS}\;\mathrm{group}-\mathrm{Compoud}\;\mathrm{group}}{\mathrm{LPS}\;\mathrm{group}-\mathrm{DMSO}\;\mathrm{group}}\times 100$$

IC_50_ was defined as the concentration of compound that inhibited 50% NO production relative to the LPS group and was calculated using SPSS 16.0 software. All assays were performed in triplicate.

### 3.5. Antimicrobial Activity 

Antimicrobial activities were evaluated by the conventional broth dilution assay [24]. Two Gram-positive—*S. aureus* (ATCC 12228) and *B. subtilis* (ATCC 6633)—and three Gram-negative—*E. coli* (ATCC 25922), *K. pneumoniae* (ATCC 13883), and *A. calcoaceticus* (ATCC 23055)—were used. Overnight cultures of five bacterial strains were made up in 0.9% saline to an inoculum density of 5 × 10^5^ cfu by comparison with a MacFarland standard. All compounds were dissolved in DMSO and diluted by Mueller Hinton broth to a starting concentration of 2 mg·mL^–1^. Ninety-five μL of MHB and 5 μL of test compounds or the antibiotic were dispensed into wells as well as the 100 μL bacterial suspension. After incubation at 37 °C for 24 h, the inhibitory effect was evaluated by optical density measurement. The MIC was determined as the concentration which the growth was inhibited 80% of bacterial. One hundred μL bacterial suspension were added to the solutions in 96-well to achieve a final volume of 200 μL and final sample concentrations from 50 to 0.125 μg·mL^–1^. The blank well was also incubated with only medium under the same conditions. OD measurement was record at 595 nm. All experiments were performed in triplicate and with ciprofloxacin and gentamicin as the positive control.

## 4. Conclusions

In conclusion, nine secondary metabolites including three new dichloroisocumarins—dichlorodiaportintone (**1**), desmethyldichlorodiaportintone (**2**), and desmethyldichloro-diaportinol (**3**)—and six known compounds—dichlorodiaportinol (**4**), desmethyldichlorodiaportin (**5**), dichlorodiaportin (**6**), mucorisocoumarin B (**7**), citroisocoumarin (**8**), and diaportinol (**9**)—were isolated from the marine mangrove-derived endophytic fungus *Ascomycota* sp. CYSK-4. Their structures were clarified by analysis of NMR data. Compounds **1**–**2** and **5**–**6** showed anti-inflammatory activities by inhibiting the production of NO in LPS-induced RAW 264.7 cells with IC_50_ values of 41.5, 15.8, 33.6, and 67.2 μM, respectively. Compounds **1**, **5**, and **6** showed inhibitory activity against *S. aureus*, *B. subtilis*, *E. coli*, *K. pneumoniae* and *A. calcoaceticus* with MIC values of 25–50 μg·mL^–1^. The results above proved that the dichloroisocumarins have the potential to be used as natural anti-inflammatory agents and antibiotics through appropriate structural modification.

## Figures and Tables

**Table 1:** ^1^H NMR (nuclear magnetic resonance) (ppm, mult, (*J* in Hz)) data of compounds **1**–**3** in acetone-*d*_6_ (500 MHz).

Position	1	2	3
4	6.68, s	6.63, s	6.69, s
5	6.62, d (2.2)	6.43, d (2.0)	6.51, d (2.1)
7	6.51, d (2.2)	6.50, d (2.0)	6.44, d (2.1)
9	3.33, d (6.0)	3.31, m	4.38, d (8.8)
10			4.28, dd (1.6, 8.8)
11*α*	3.04, dd (9.5, 14.7)	3.04, dd (9.5, 14.7)	6.43, d (1.6)
11*β*	2.45, dd (6.9, 14.7)	2.45, dd (6.8, 14.4)	
12	4.75, dd (6.9, 9.5)	4.75, dd (6.9, 9.3)	
14	6.52, s	6.52, s	
6-OCH_3_	3.92, s		
8-OH	11.0, br s	11.0, br s	10.9, br s

**Table 2:** ^13^C NMR (ppm, mult) data of compounds **1–3** in acetone-*d*_6_ (125 MHz).

Position	1	2	3
1	166.5, C	166.5, C	166.6, C
3	151.7, C	151.5, C	156.1, C
4	109.8, CH	109.7, CH	107.5, CH
4a	139.9, C	140.1, C	140.1, C
5	102.6, CH	102.9, CH	104.3, CH
6	167.9, C	166.4, C	164.6, C
7	101.6, CH	104.1, CH	103.0, CH
8	164.2, C	164.4, C	166.6, C
8a	100.9, C	100.1, C	100.3, C
9	41.2, CH_2_	41.2, CH_2_	73.1, CH
10	86.0, C	86.0, C	76.7, CH
11	37.4, CH_2_	37.4, CH_2_	76.1, CH
12	68.4, CH	68.4, CH	
13	175.6, C	175.5, C	
14	77.2, CH	77.2, CH	
6-OCH_3_	56.3, CH_3_		

**Table 3:** Inhibitory effects of isolated compounds on lipopolysaccharide (LPS)-stimulated nitric oxide (NO) production in RAW (mice macrophage) 264.7 cells.

Compound	1	2	3	4	5	6	7	8	9	Indometacin ^a^
IC_50_ (μM)	41.5	15.8	>100	>100	33.6	67.2	>100	>100	>100	37.5

^a^ Positive control.

**Table 4:** Antibacterial activities of compounds **1**–**9**.

Compound ^a^	MIC (μg·mL^−1^)				
	*Staphylococcus aureus*	*Bacillus subtilis*	*Escherichia coli*	*Klebsiella pneumoniae*	*Acinetobacter calcoaceticus*
1	50	>50	50	50	>50
5	25	25	25	25	50
6	25	25	50	50	50
Ciprofloxacin ^b^	0.25	0.50	0.50	0.25	0.25
Gentamicin ^b^	0.10	0.25	0.25	0.25	0.25

^a^ Compounds **2**, **3**, **4**, **7**, **8**, and **9** showed no activities (MIC > 50 μg·mL^–1^); ^b^ Positive control.

## Supplementary Materials

The following are available online at http://www.mdpi.com/1660-3397/16/2/54/s1, Figure S1. ^1^H NMR spectrum of compound **1** (500 MHz, acetone-*d*_6_), Figure S2. ^1^H NMR spectrum of compound **1** (500 MHz, DMSO-*d*_6_), Figure S3. ^13^C NMR spectrum of compound **1** (125 MHz, acetone-*d*_6_), Figure S4. HSQC spectrum of compound **1** (acetone-*d*_6_), Figure S5. HMBC spectrum of compound **1** (acetone-*d*_6_), Figure S6. ^1^H-^1^H COSY spectrum of compound **1** (acetone-*d*_6_), Figure S7. NOESY spectrum of compound **1** (acetone-*d*_6_), Figure S8. NOESY spectrum of compound **1** (DMSO-*d*_6_), Figure S9. HRESIMS spectrum of compound **1**, Figure S10. ^1^H NMR spectrum of compound **2** (500 MHz, acetone-*d*_6_), Figure S11. ^13^C NMR spectrum of compound **2** (125 MHz, acetone-*d*_6_), Figure S12. HSQC spectrum of compound **2** (acetone-*d*_6_), Figure S13. HMBC spectrum of compound **2** (acetone-*d*_6_), Figure S14. ^1^H-^1^H COSY spectrum of compound **2** (acetone-*d*_6_), Figure S15. NOESY spectrum of compound **2** (acetone-*d*_6_), Figure S16. HRESIMS spectrum of compound **2**, Figure S17. ^1^H NMR spectrum of compound **3** (500 MHz, acetone-*d*_6_), Figure S18. ^1^H NMR spectrum of compound **3** (500 MHz, DMSO-*d*_6_), Figure S19. ^13^C NMR spectrum of compound **3** (125 MHz, acetone-*d*_6_), Figure S20. HSQC spectrum of compound **3** (acetone-*d*_6_), Figure S21. HMBC spectrum of compound **3** (acetone-*d*_6_), Figure S22. ^1^H-^1^H COSY spectrum of compound **3** (acetone-*d*_6_), Figure S23. NOESY spectrum of compound **3** (acetone-*d*_6_), Figure S24. NOESY spectrum of compound **3** (DMSO-*d*_6_), Figure S25. HRESIMS spectrum of compound **3**, Figure S26. Experiment ECD spectrum of **1** and **2**.
