# Supplementary material for: Dichloroisocoumarins with Potential Anti-Inflammatory Activity from the Mangrove Endophytic Fungus Ascomycota sp. CYSK-4
Source: Mar Drugs. 2018 Feb 9;16(2):54. doi: 10.3390/md16020054 (PMC5852482; doi:10.3390/md16020054)
Supplement: Supplementary file 1 [file marinedrugs-16-00054-s001.docx]

**Supplementary Materials: Dichloroisocoumarins with potential anti-inflammatory activity from the mangrove endophytic fungus *Ascomycota* sp. CYSK-4**

Yan Chen ^1^, Zhaoming Liu ^2^, Hongju Liu ^2, 3^, Yahong Pan ^2^, Jing Li ^3^, Lan Liu ^1,*,^ and Zhigang She ^1,2,*^

^1^ School of Marine Sciences, Sun Yat-sen University; South China Sea Bio-Resource Exploitation and Utilization Collaborative Innovation Center, Guangzhou 510006, China; [chenyan27@mail2.sysu.edu.cn](mailto:chenyan27@mail2.sysu.edu.cn) (C.Y.);

^2^ School of Chemistry, Sun Yat-Sen University, Guangzhou 510275, P. R. China; [liuzhaom@mali2.sysu.edu.cn](mailto:liuzhaom@mali2.sysu.edu.cn) (Z.L.); [liuhj8@mail2.sysu.edu.cn](mailto:liuhj8@mail2.sysu.edu.cn) (H.L.); [pan16a@126.com](mailto:pan16a@126.com) (Y.P.);

^3^ School of Pharmacy, Guangdong Medical University, Dongguan, 523808, China; [lijinggdmu@yahoo.com](mailto:lijinggdmu@yahoo.com) (J.L.);

***** Correspondence: [cesllan@mail.sysu.edu.cn](mailto:cesllan@mail.sysu.edu.cn) (L.L.); [cesshzhg@mail.sysu.edu.cn](mailto:cesshzhg@mail.sysu.edu.cn) (Z.S.);

Tel.: +86-20-8472-5459 (L. L.); +86-20-8411-3356 (Z. S.);


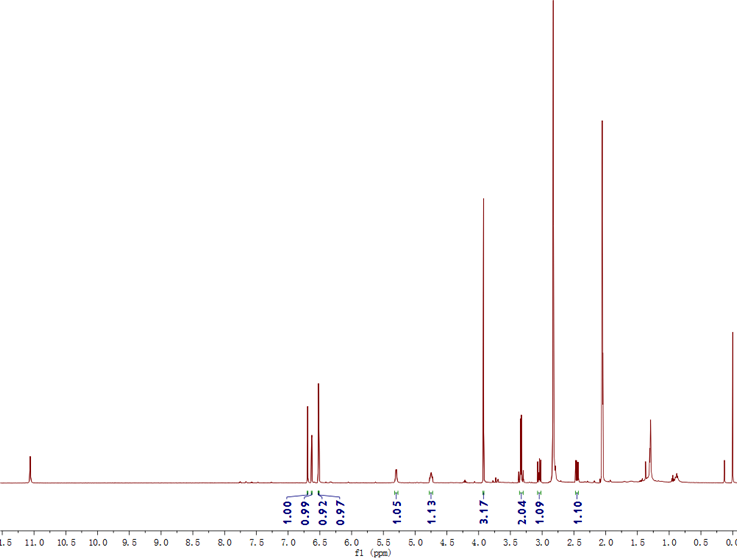


**Figure S1.** ^1^H NMR spectrum of compound **1** (500 MHz, acetone-*d*_6_).


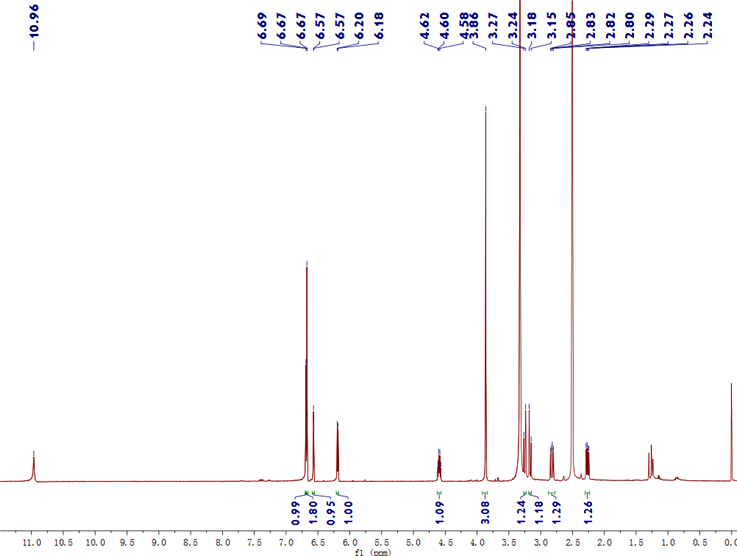


**Figure S2.** ^1^H NMR spectrum of compound **1** (500 MHz, DMSO-*d*_6_)


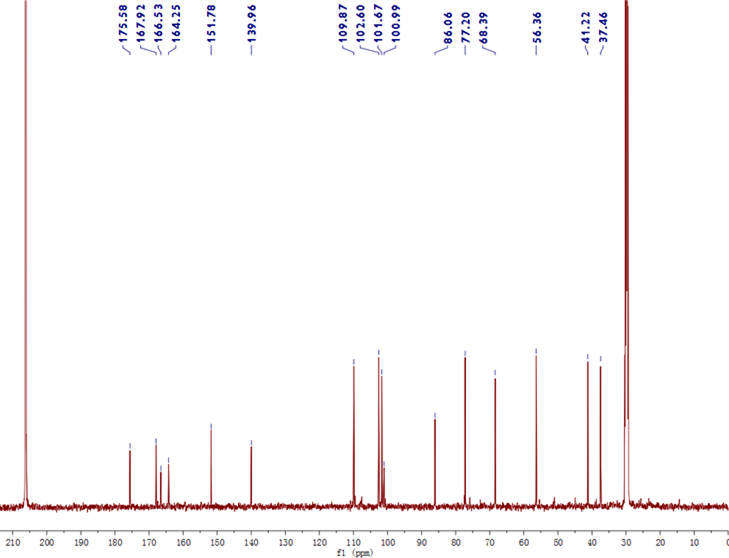


**Figure S3.** ^13^C NMR spectrum of compound **1** (125 MHz, acetone-*d*_6_).


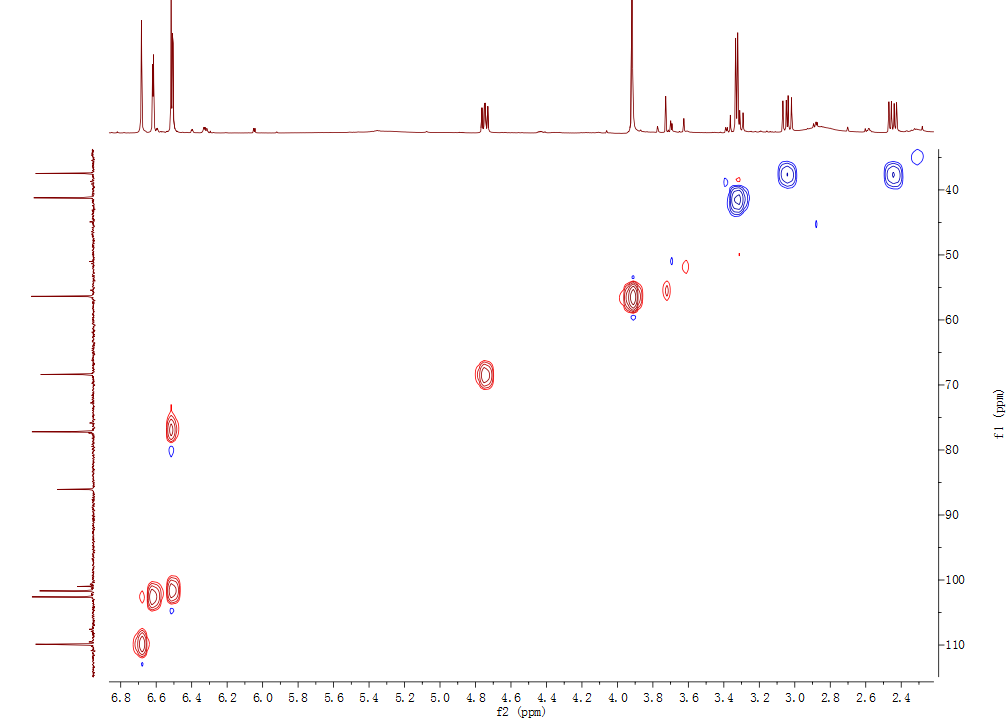


**Figure S4.** HSQC spectrum of compound **1** (acetone-*d*_6_).


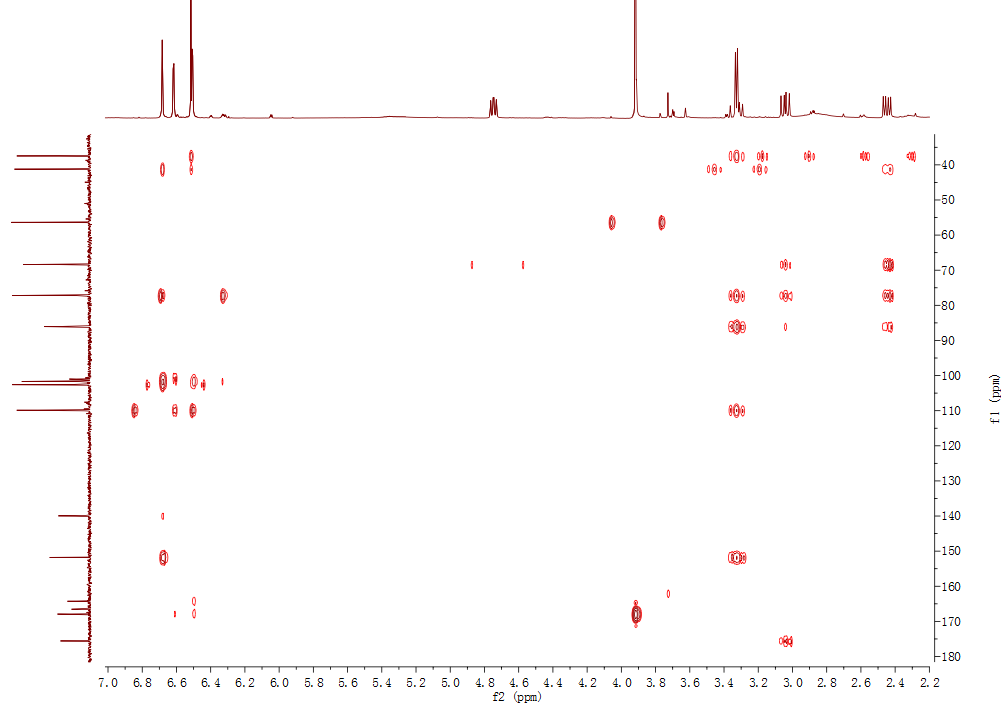


**Figure S5.** HMBC spectrum of compound **1** (acetone-*d*_6_).


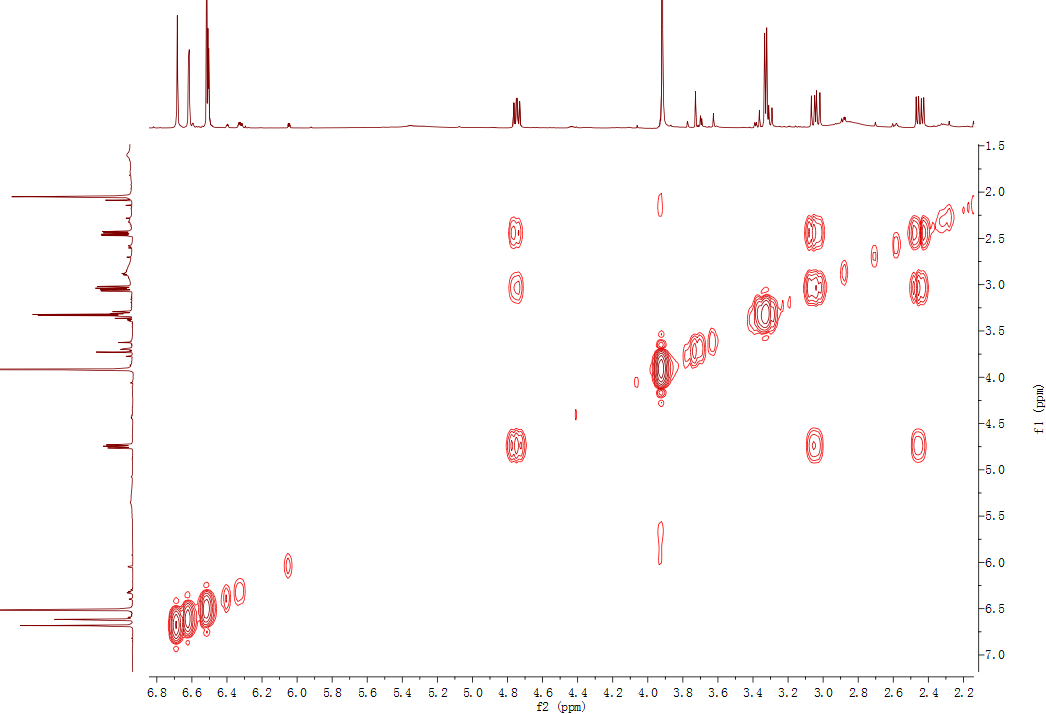


**Figure S6.** ^1^H-^1^H COSY spectrum of compound **1** (acetone-*d*_6_).


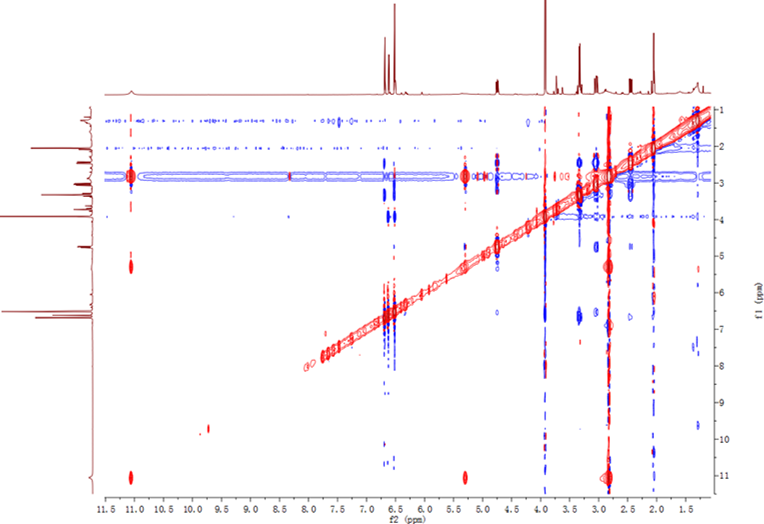


**Figure S7.** NOESY spectrum of compound **1** (acetone-*d*_6_).


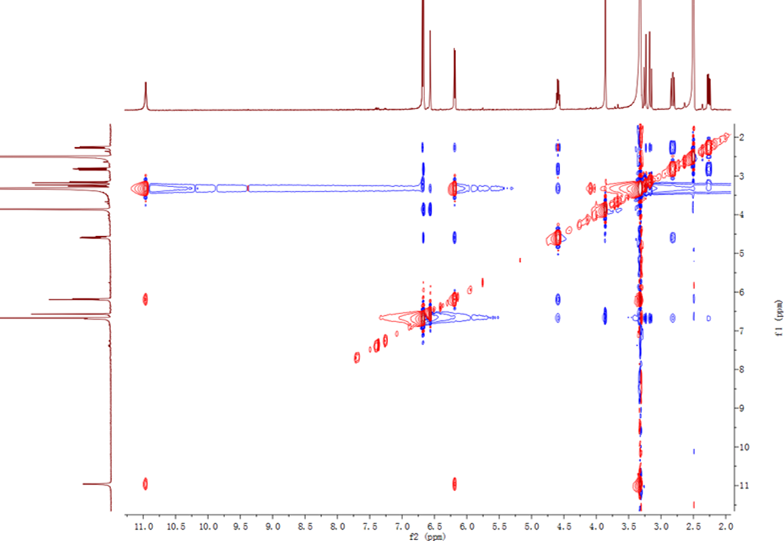


**Figure S8.** NOESY spectrum of compound **1** (DMSO-*d*_6_).


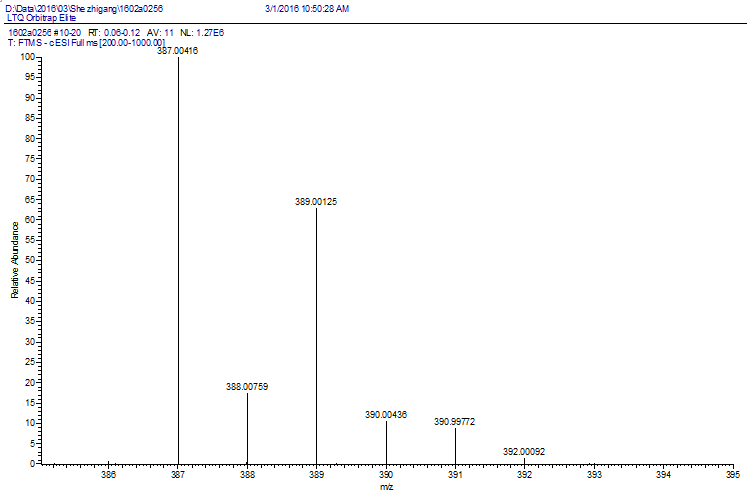


**Figure S9.** HRESIMS spectrum of compound **1**.


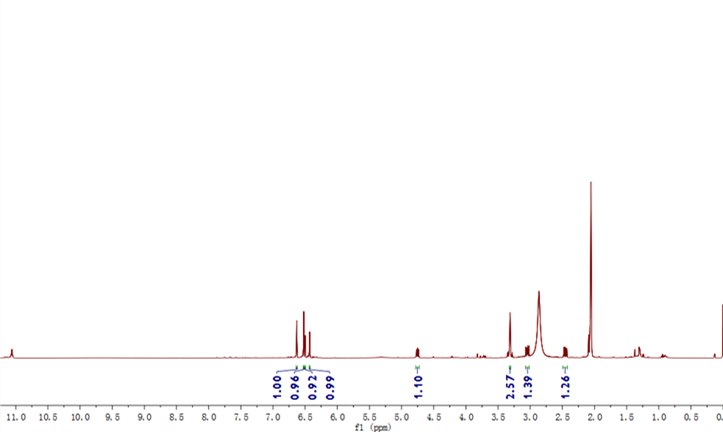
**Figure S10.** ^1^H NMR spectrum of compound **2** (500 MHz, acetone-*d*_6_).


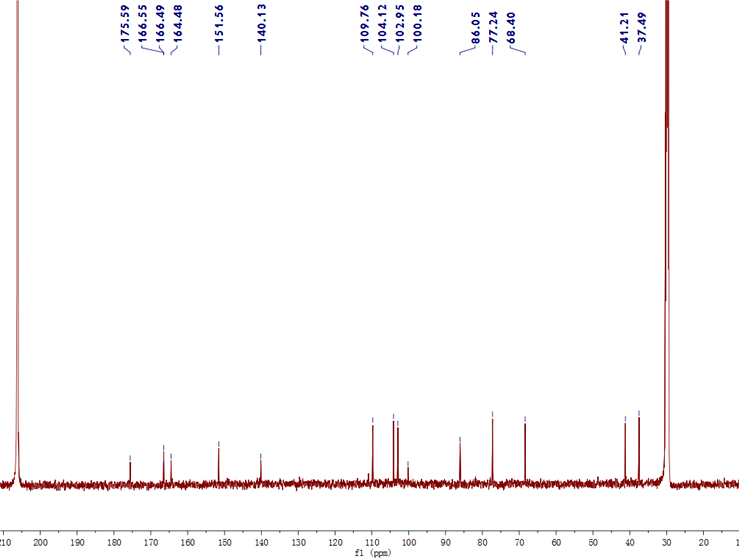
**Figure S11.** ^13^C NMR spectrum of compound **2** (125 MHz, acetone-*d*_6_).


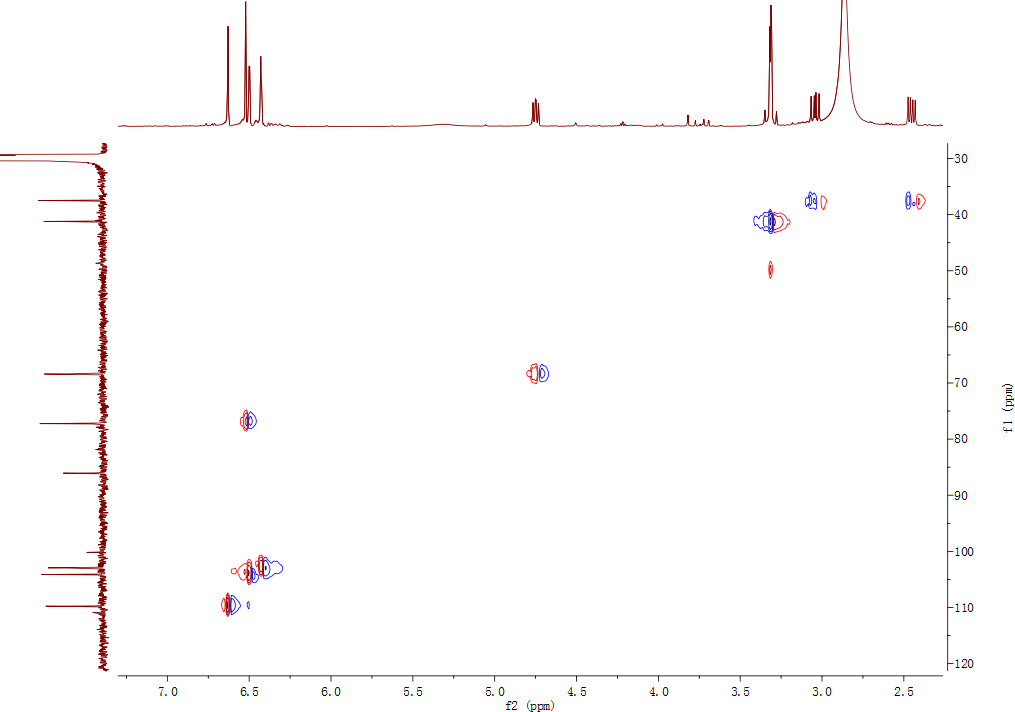


**Figure S12.** HSQC spectrum of compound **2** (acetone-*d*_6_).


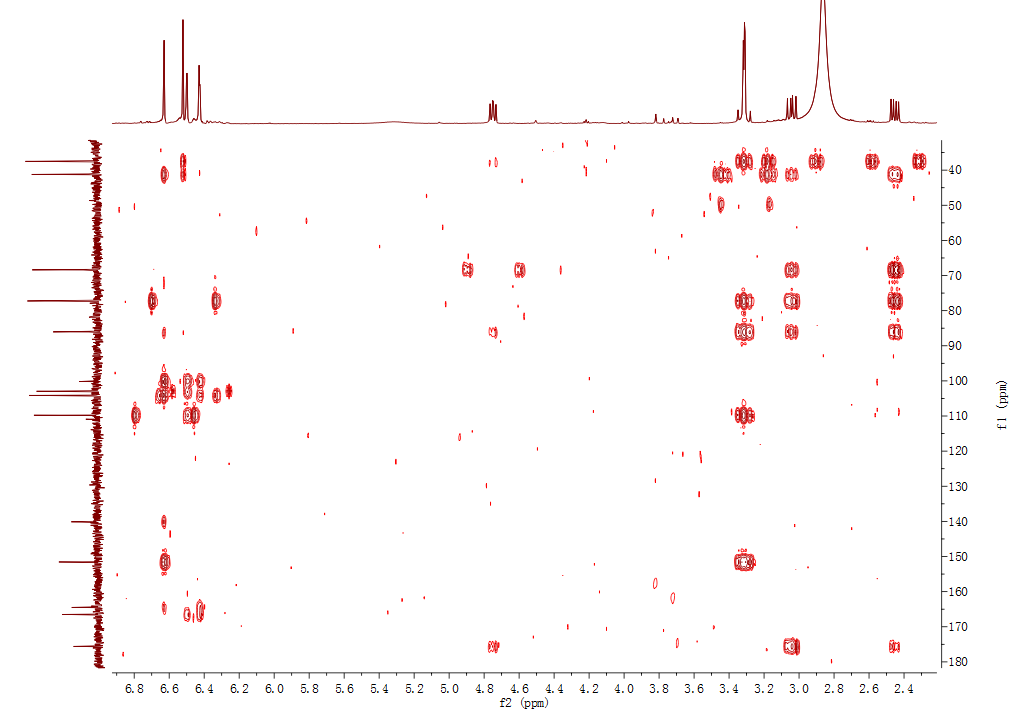


**Figure S13.** ^1^H MBC spectrum of compound **2** (acetone-*d*_6_).


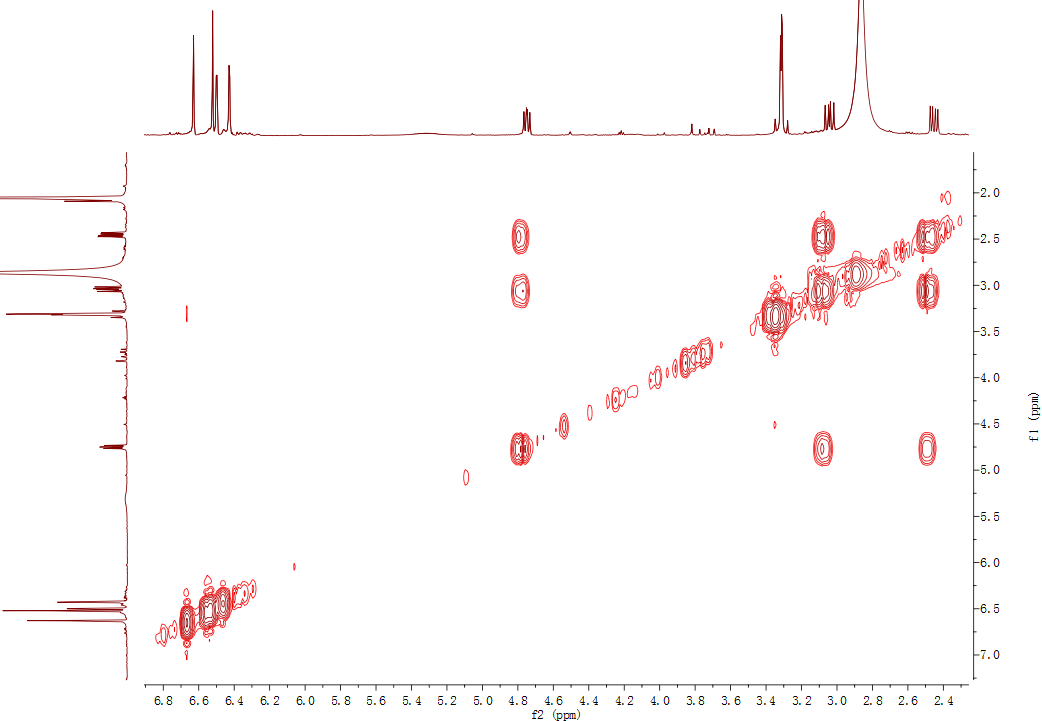


**Figure S14.** ^1^H-^1^H COSY spectrum of compound **2** (acetone-*d*_6_).


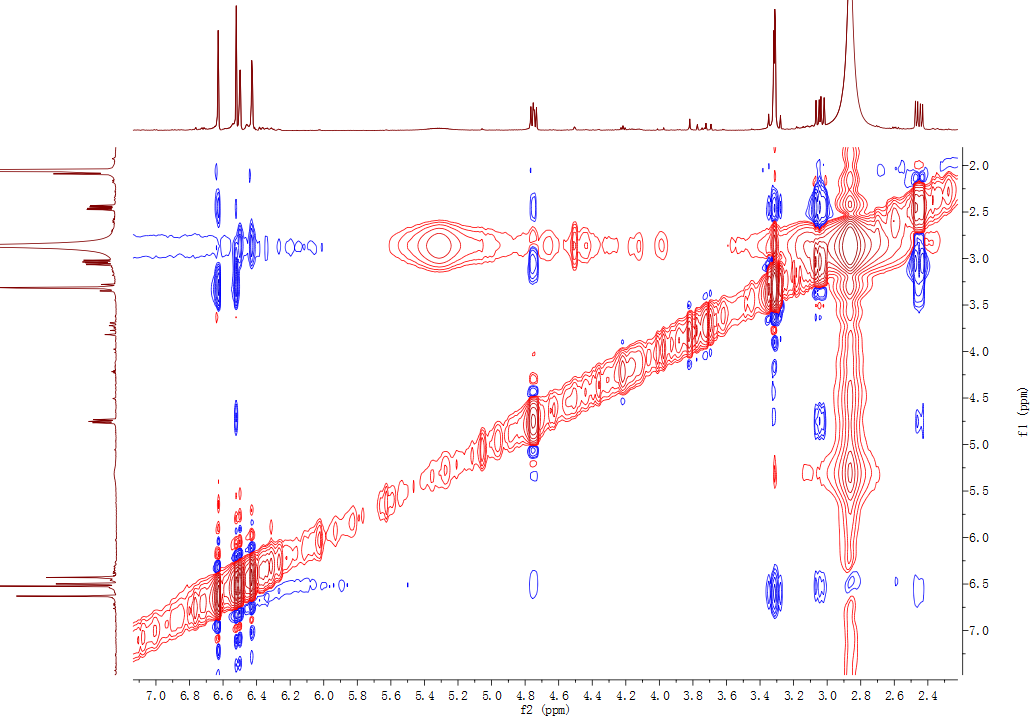
**Figure S15.** NOESY spectrum of compound **2** (acetone-*d*_6_).


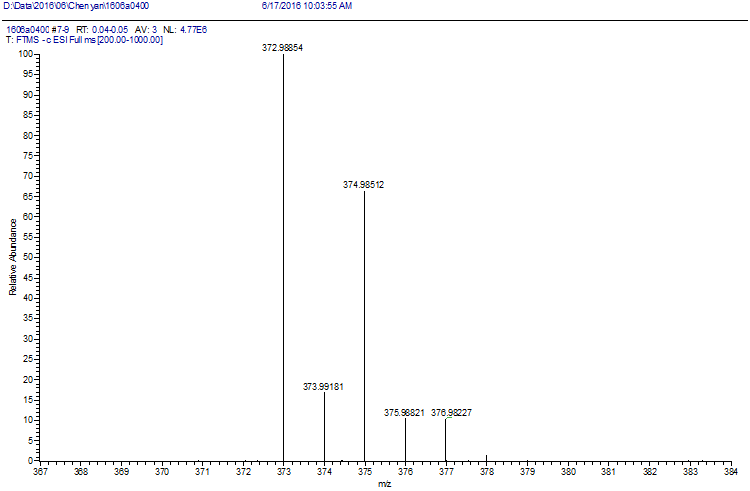


**Figure S16.** HRESIMS spectrum of compound **2**.


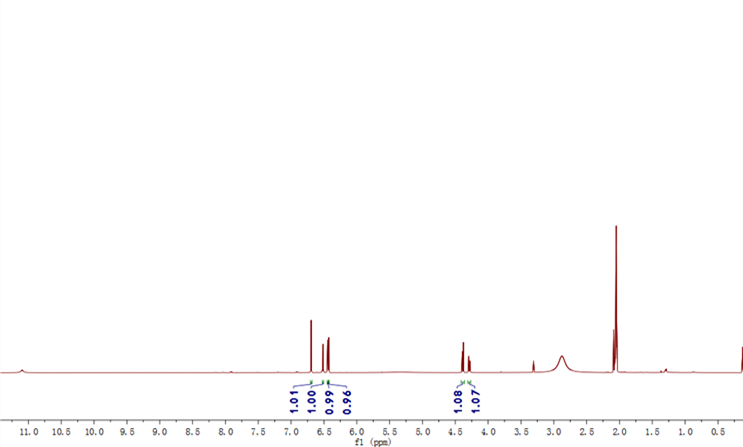


**Figure S17.** ^1^H NMR spectrum of compound **3** (500 MHz, acetone-*d*_6_).


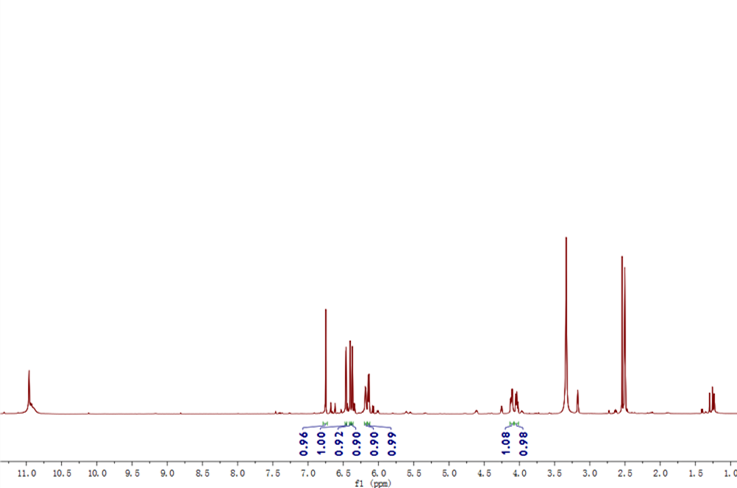


**Figure S18.** ^1^H NMR spectrum of compound **3** (500 MHz, DMSO-*d*_6_)


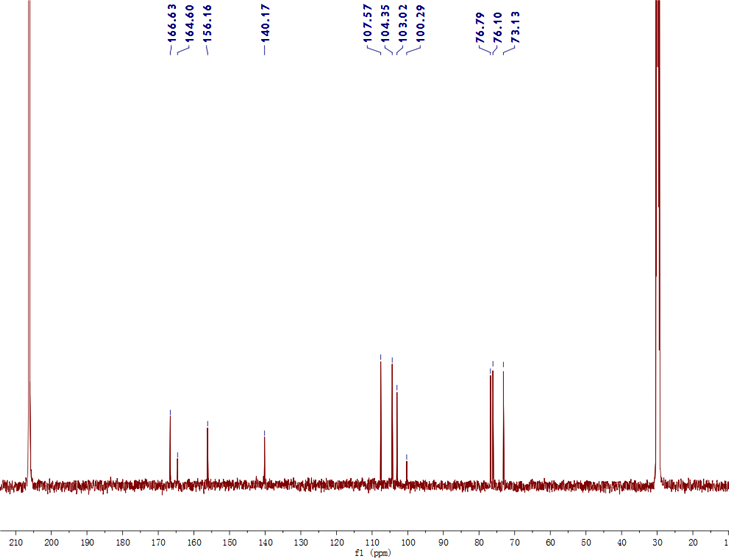


**Figure S19.** ^13^C NMR spectrum of compound **3** (125 MHz, acetone-*d*_6_).


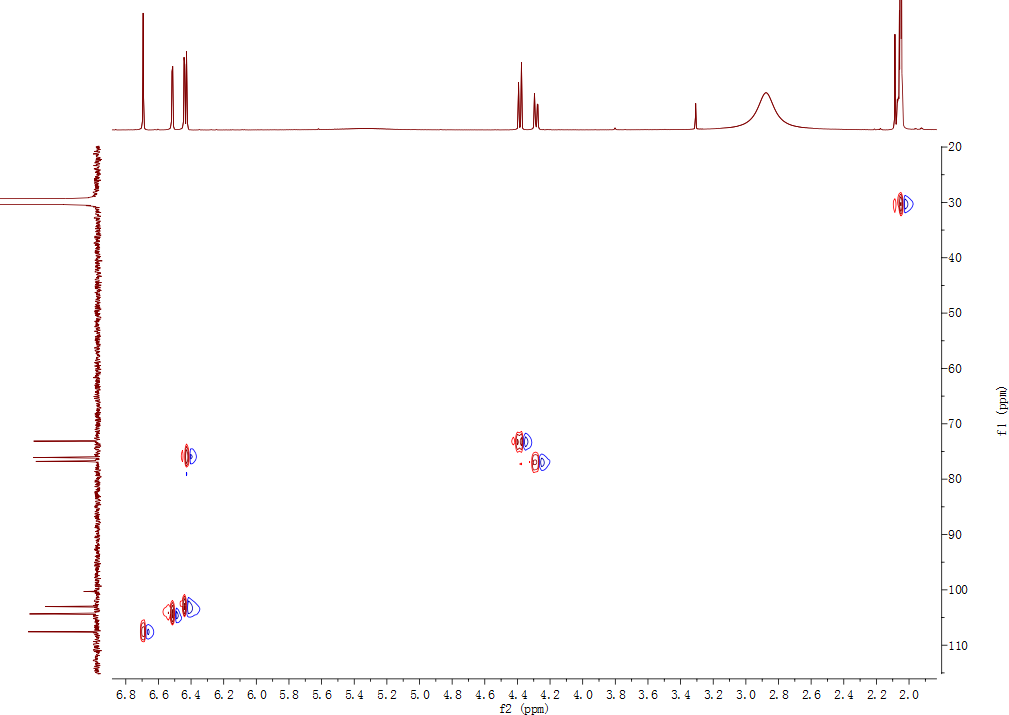


**Figure S20.** HSQC spectrum of compound **3** (acetone-*d*_6_).


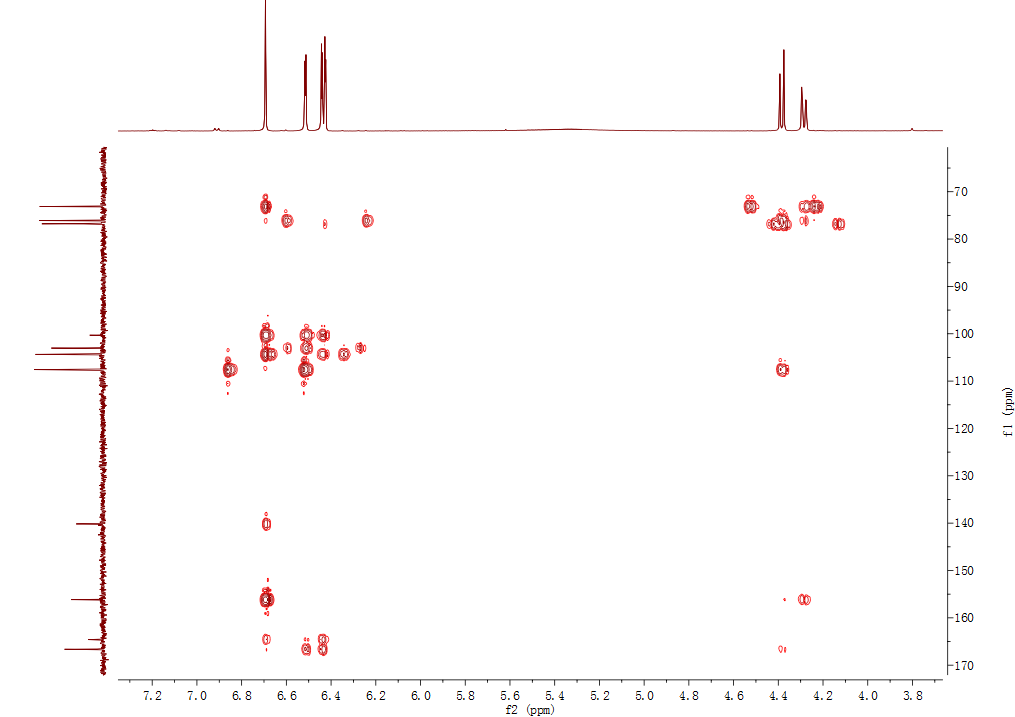


**Figure S21.** HMBC spectrum of compound **3** (acetone-*d*_6_).


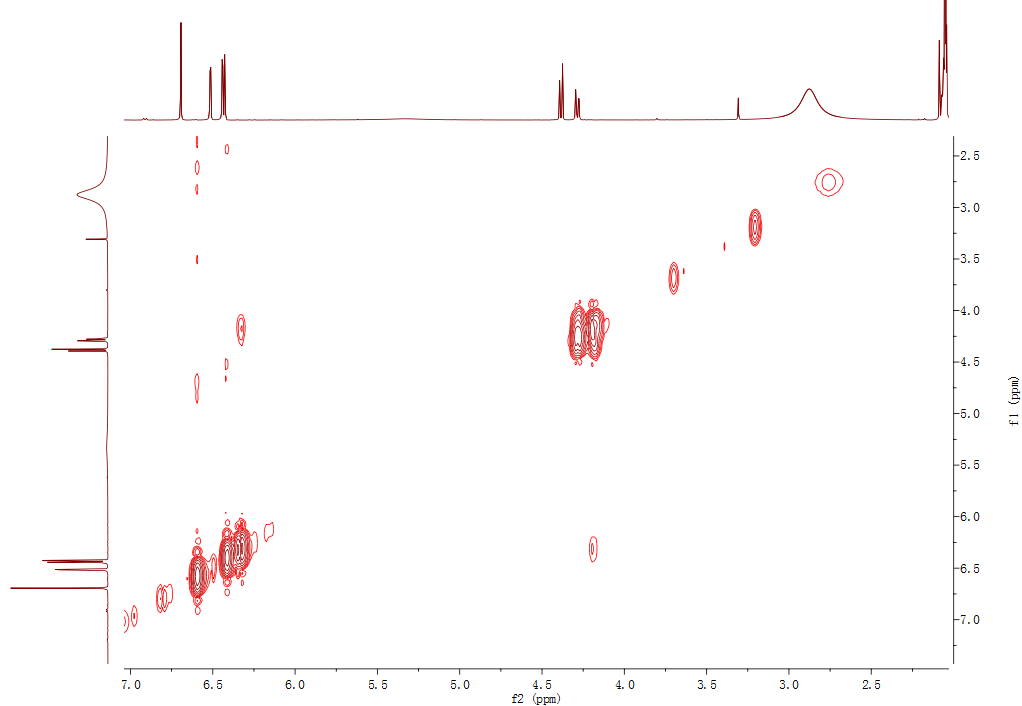


**Figure S22.** ^1^H-^1^H COSY spectrum of compound **3** (acetone-*d*_6_).


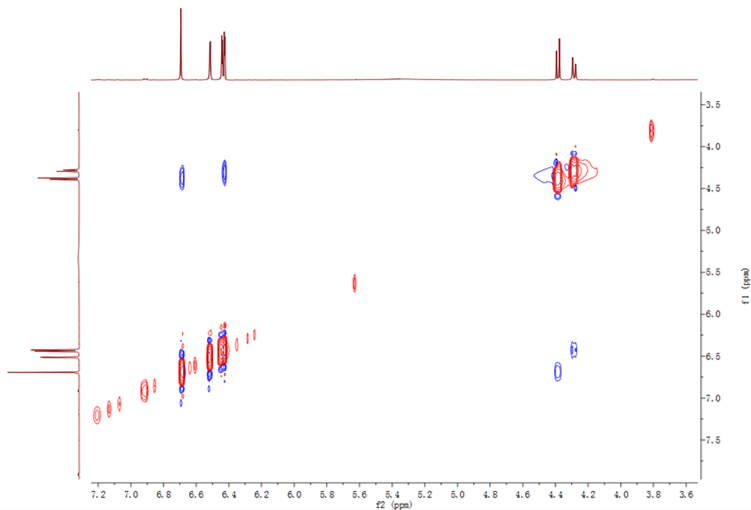


**Figure S23.** NOESY spectrum of compound **3** (acetone-*d*_6_).


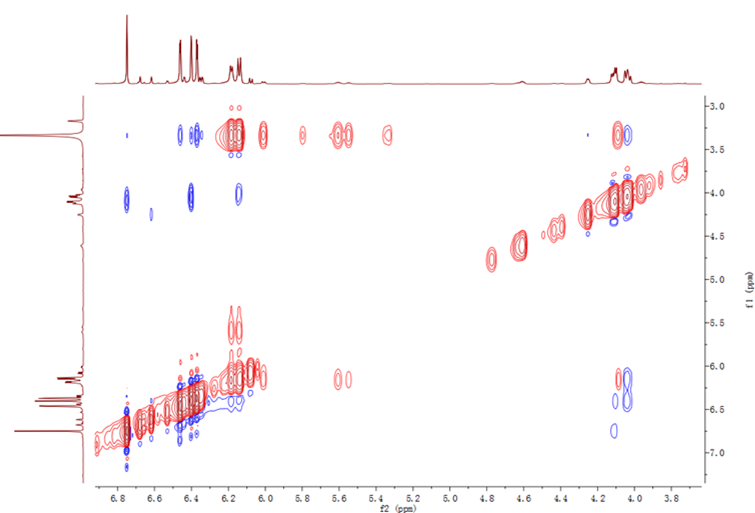


**Figure S24.** NOESY spectrum of compound **3** (DMSO-*d*_6_).


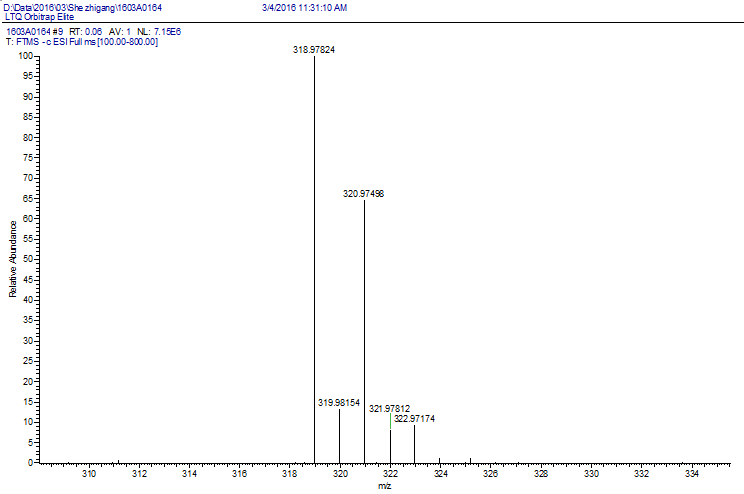
**Figure S25.** HRESIMS spectrum of compound **3**.


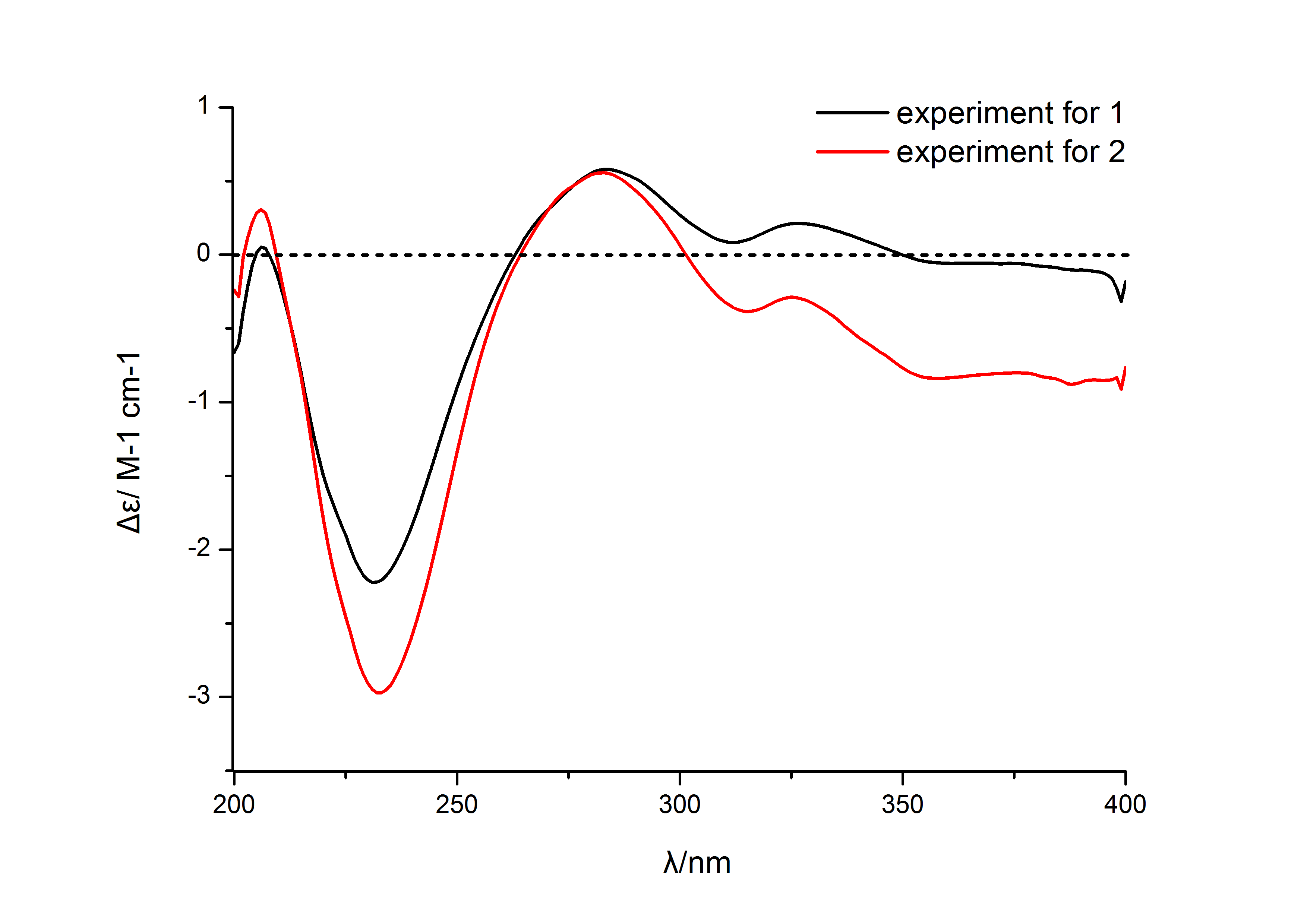


**Figure S26.** Experiment ECD spectra of **1** and **2**.

**Gene of the fungus :** CTTCCGTAAGGGTAACCTGCGGAAGGATCATTACCTAGAGTTGCGGGC TTTGCCTGcCATCTCTTACCCATGTCTTTTGAGTACCTTCGTTTCCTCGGCGGGTTCGCCCGCCGGTTGGACAACACTTAAACCCTTTGTAATTGAAATCAGCGTCTGAAAAAACTTTAATAGTTACAACTTTCAACAACGGATCTCTTGGTTCTGGCATCGATGAAGAACGCAGCGAAATGCGATAAGTAGTGTGAATTGCAGAATTCAGTGAATCATCGAATCTTTGAACGCACATTGCGCCCCTTGGTATTCCATGGGGCATGCCTGTTCGAGCGTCATTTGTACCTTCAAGCTCTGCTTGGTGTTGGGTGTTTGTCTCGCCTCTGCGCGCAGACTCGCCTCAAAGCAATTGGCAGCCGGCGTATTGATTTCGGAGCGCAGTACATCTCGCGCTTTGCACTCATAACGACGACGTCCAAAAAGTACATTTTTTACACTCTtGACCTCGgATCAGGTAGGGATACCCGCTGAACTTAAGCATATCAATAGCCGGGAGGAAA

The sequence of the fungus *Ascomycota* sp. was carried out as described below. About 120 mg of fresh fungal mycelium was collected in a microcentrifuge tube (1.5 mL) to extract genomic DNA from the fungus using a fungal DNA kit (50) (E.Z.N.A., Omega) according to the manufacturer’s protocol. The PCR reactions were performed in a ﬁnal volume of 50 *μ*L, which was composed of template DNA (2 *μ*L), 5 *μ*L of 10× buﬀer, 1 *μ*L of dNTP, 0.5 *μ*L of ITS1F, 0.5 *μ*L of ITS4 (20 *μ*mol/mL each), 0.25 *μ*L of Taq polymerase, and appropriate ultrapure water under the following conditions: (1) initial denaturation at 94.0 °C for 5 min; (2) desmolysis at 94.0 °C for 50 s; (3) annealing at 52.5 °C for 50 s; (4) extension at 72.0 °C for 1 min; (5) ﬁnal extension at 72.0 °C for 10 min. Steps 2−4 were repeated 32 times. Then, 5 *μ*L of the ampliﬁcation products was loaded on an agarose gel (1.2% agarose in 0.5× TAE, 5 μL of ethidium bromide 1% m/v solution per 100 mL of gel). After electrophoresis at 100 V for 40 min, the band due to the PCR product (approximate size 600 bp) was isolated from the gel slice using a gel extraction kit (E.Z.N.A., Omega) according to the manufacturer’s protocol. The PCR product was then submitted for sequencing (Invitrogen, Shanghai, China) with the primer ITS1F.
